# A Budget Impact Model for the use of Drug-Eluting Stents in Patients with Symptomatic Lower-Limb Peripheral Arterial Disease: An Australian Perspective

**DOI:** 10.1007/s00270-021-02848-8

**Published:** 2021-06-21

**Authors:** Nishath Altaf, Thathya Venu Ariyaratne, Adrian Peacock, Irene Deltetto, Jad El-Hoss, Shannon Thomas, Colman Taylor, Bibombe Patrice Mwipatayi

**Affiliations:** 1 Department of Vascular Surgery, Royal Perth Hospital, Royal Perth Bentley Group, Level 6, North Block, Wellington Street, Perth, WA 6000 Australia; 2 Boston Scientific Corporation, Sydney, NSW Australia; 3 Health Technology Analysts, Sydney, NSW Australia; 4 Department of Vascular Surgery, Prince of Wales Hospital, Sydney, NSW Australia; 5 The George Institute for Global Health, Sydney, NSW Australia; 6 The University of NSW, Sydney, NSW Australia

**Keywords:** Peripheral arterial disease, Budget impact model, Target lesion revascularisation, Paclitaxel-eluting stent, Superficial femoral artery, Healthcare system costs

## Abstract

**Purpose:**

Improvement in long-term outcomes through innovative, cost-effective medical technologies is a focus for endovascular procedures aimed at treating symptomatic lower-limb peripheral arterial disease (PAD). The advent of drug-eluting stents (DES) has improved symptomatic PAD treatment via a reduction in high rates of target lesion revascularisation (TLR). The present study aimed to compare the 5-year financial impact of treatment with Eluvia, a new paclitaxel-eluting stent, versus treatment with Zilver PTX, a drug-coated stent, among patients in Australia by developing a budget impact model (BIM).

**Methods:**

A BIM was developed from an Australian public hospital payer perspective using Australian national cost weights (AUD), published literature, and public hospital audit data. Clinical outcomes, including clinically driven TLRs (CD-TLRs), adverse events, and length of stay, were based on the 2-year results of the IMPERIAL trial, which compared Eluvia DES to Zilver PTX.

**Results:**

Assuming EVP eligibility rate of 80% and DES uses rate ranging from 10 to 28% (superficial femoral artery lesions only), the 5-year model forecasted a treatment population between 14,428 and 40,399 patients. The model estimated 1499–4198 fewer CD-TLRs and 16,515–46,243 fewer hospital days with Eluvia DES use. This translated to 5-year potential savings of $4.3–$12.1 million to the Australian public hospital payer attributable to reduced CD-TLRs for Eluvia DES and $33.1–$92.6 million to Australian public hospitals owing to reduced adverse events and hospital bed days.

**Conclusion:**

Eluvia DES use as treatment for symptomatic lower-limb PAD could lead to potential savings for the Australian public healthcare system based on improved patient outcomes.

**Supplementary Information:**

The online version contains supplementary material available at 10.1007/s00270-021-02848-8.

## Introduction

Peripheral arterial disease (PAD) management continues to evolve with the advent of improvements in technology. Consequences of financial and resource utilisation to the health payer are often overlooked. In a value-driven, hyperopic healthcare system, the superior efficacy and safety outcomes that further reduce resource utilisation and thereby promote additional economically relevant outcomes can result in considerable cost savings to the healthcare payer.

The introduction of Zilver PTX (Cook Medical, Bloomington, IN, USA), a non-polymer-based paclitaxel-coated nitinol stent, represented a significant clinical innovation for endovascular therapies a decade ago, leading to improved patency rates and reduced rates of target lesion revascularisation (TLR) as compared to those with bare metal stents (BMS) and drug-coated balloons, respectively [1, 2].

Recently, a primary patency rate of 96.4% at 12 months was reported for Eluvia (Boston Scientific, Marlborough, MA, USA), a novel paclitaxel-eluting stent with more sustained drug elution over a longer period [3]. The IMPERIAL randomised controlled trial compared the safety and efficacy of the Eluvia paclitaxel-eluting stent with those of the Zilver PTX drug-coated stent in treating lesions in the femoropopliteal artery [4–6]. At 12 months, the primary patency in the Eluvia arm (86.8%, 231/266) was superior to that in the Zilver PTX arm (81.5%, 106/130; *p* < 0.001) [4]. Notably, at 12 months, the proportion of patients who underwent CD-TLR in the Eluvia group was half of that in the Zilver PTX group (4.5%, 13/287 vs. 9%, 13/145; *p* = 0.067) [4]. At 24 months, the significance of this outcome was confirmed in the Eluvia group, which exhibited a 42% reduction in CD-TLRs as compared to the Zilver PTX group (12.7% vs. 20.1%; *p* = 0.049)[5, 6].

The Eluvia drug-eluting stent (DES) was first introduced in 2017 to Australian clinical practice, where medical device utilisation in the resource-constrained public healthcare system is based on tender-driven contracts between manufacturers and public hospitals. Public hospitals are, in turn, funded by state governments through activity-based funding. While value-based procurement is not widely prevalent in the Australian public healthcare system, evidence of cost savings via improvements in patient outcomes is relevant to stakeholders at all layers of the healthcare system.

The present study aimed to compare the 5-year financial impact of treatment with Eluvia DES versus treatment with Zilver PTX drug-coated stent among Australian patients with PAD lesions in the superficial femoral artery (SFA) by developing a budget impact model (BIM).

## Materials and Methods

### Model Structure

The BIM was constructed based on published international health economic guidelines for BIM development [7]. Australian patients with symptomatic PAD lesions in the SFA who were not eligible for or had not responded to treatments such as lifestyle modifications, exercise regimens, or pharmacological interventions were included in the model cohort. The impact of Eluvia DES use versus Zilver PTX drug-coated stent use was evaluated at both a national and state level. This model also enabled the comparison of outcomes at the individual hospital or local health district level. Potential budget savings or gains were expressed as the difference in costs between a scenario in which only Zilver PTX and only Eluvia DES was used.

### Perspective and Time Horizon

The primary perspective of the BIM was that of the Australian public healthcare system. In Australia, public hospitals receive funding from state budgets based on the number and mix of patients they treat. Hospitals receive a predetermined amount for a case, irrespective of how long they stay in hospital. This incentivises hospitals to save money through reducing length of stay [8]. Therefore, an additional ‘hospital perspective’ was included to capture potential cost savings not observed through a pure activity-based funding perspective (‘Australian public healthcare system’). The model considered a 5-year horizon commencing in 2019, whereby the outcomes of the IMPERIAL trial at 12 and 24 months as well as the related costs were applied to each annual patient cohort.

### Epidemiology, Clinical Outcomes and Resource Utilisation

A funnel approach employing national epidemiological data, published literature, and clinical expert input (Table 1; detailed in Supplement) was used to estimate the base-case patient cohort, using data from published sources as well as data from Australian hospital audits [1, 4, 9, 10]. Annual procedure growth rate was informed by the mean yearly increase of the number of stent procedures in Australia over the years 2014–2017 [11]. From a resource-use perspective, critical outcomes of PAD treatment pertain to restenosis and subsequent hospital readmissions for adverse events (AEs) were considered, including CD-TLRs and major limb amputations. The model used all primary major AE outcomes from the 12-month and 24-month IMPERIAL trial data (Table 2), to estimate event rates in the cohort, and their direct costs related to treatment with Eluvia DES and Zilver PTX. Patients requiring TLR had undergone one of the following procedures: percutaneous transluminal angioplasty (PTA) without stenting, PTA with single-stent placement, PTA with two-stent placement, or open surgical intervention [12]. The number of hospital days due to AEs observed in the IMPERIAL trial was also considered in the model to estimate hospital-related costs (Table 2). In the IMPERIAL trial, hospital days were recorded and reported for patients who were admitted due to AEs rather than for the primary intervention [4].

**Table 1:**
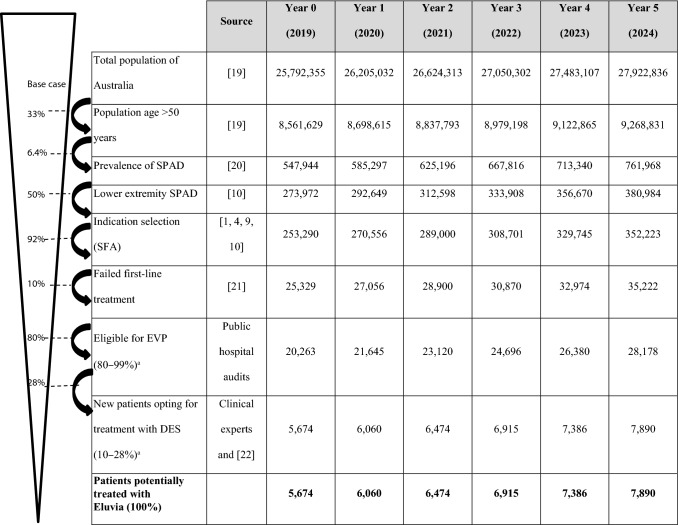
Number of patients receiving Eluvia DES in each year cohort using a funnel approach*

Values in bold were included in the base-case analysis

*DES* drug-eluting stent, *EVP* endovascular procedure, *SFA* superficial femoral artery, *SPAD* symptomatic peripheral arterial disease

*See online Supplement for a detailed explanation of the funnel approach

^a^The ranges were tested when sensitivity analyses were performed

**Table 2:** Primary safety and key clinical outcomes and resource-use parameters from the IMPERIAL trial included in the model [4, 6]

	12 months^a^			24 months^b^		
	Eluvia (*n* = 309)	Zilver PTX (*n* = 156)	*p*-value	Eluvia (*n* = 309)	Zilver PTX (*n* = 156)	*p*-value
Major AEs						
CD-TLR	4.5% (13/287)	9.0% (13/145)	0.067	12.7% (35/275)	20.1% (27/134)	0.049
Percutaneous intervention	96.7%	96.0%	1.00	95.7%	97.1%	1.00
Surgery (open)	3.3%	4.0%	1.00	4.3%	2.9%	1.00
Major amputation of the target limb	0.3% (1/287)	0% (0/145)	1.00	1.5% (4/275)	0.7% (1/134)	> 0.99
Hospitalisation outcomes						
Hospital days for all AE^c^	13.9 (123/309)	17.7 (60/156)	> 0.05	21.5 (169/309)	18.9 (81/156)	0.620

*AEs* adverse events, *CD-TLR* clinically driven target lesion revascularisation

^a^Adapted from Table 3 and Table A6 of Gray et al. [4]

^b^Adapted from Müller-Hülsbeck, Benko [6] and data on file (hospitalisation outcomes)

^c^The average length of hospital stay for AE was calculated as 5.5 days for Eluvia DES group and 6.8 days for Zilver PTX group at 12 months (where average hospital stay = hospital days for AE * *n*/*N*), representing a 19% reduction for eluvia DES group at 12 months

### Costs

Cost inputs applied in the model are presented in Table 3. All cost inputs in this BIM were obtained from published sources [13] (see Supplement) and were measured in Australian dollars (AUD), where 1 AUD is approximately equivalent to 0.69 USD and 0.61 EUR (June 2020).

**Table 3:** Cost* inputs applied in the budget impact model

Description	Value	Reference
Cost of percutaneous endovascular intervention	$5495	[13]
Cost of surgical (open) intervention	$16,411	[13]
Cost of amputation	$35,354	[13]
Cost per day of hospital stay	$2003	[13, 23]

*The price of Eluvia and Zilver PTX stents (difference = $0) were referenced from the 2019 Australian Prostheses List (Part A) available at https://www.health.gov.au/resources/publications/prostheses-list

#### Healthcare system costs

Healthcare system costs are those incurred by state governments through activity-based funding. All direct costs related to primary procedure and event rates as observed in the IMPERIAL trial over a 24-month period were included in the model, irrespective of statistical significance. To assess expenditure from a healthcare system perspective, unit costs related to initial and subsequent hospitalisations were identified from relevant AR-DRG codes and cost weights as determined by the Independent Hospital Pricing Authority (Supplementary Table A1).

#### Hospital costs

The impact of hospital readmissions for adverse events (hospital days) was assessed using the national weighted average cost per hospital day as these hospital-level savings are not directly captured within DRG costs (Supplementary Table A2) [13].

### Sensitivity Analysis

Sensitivity analysis was conducted to assess the impact of uncertainties in key model inputs and several scenarios. These included variations in population inputs, including the rates of patients eligible for endovascular procedures (EVP; 70–99%) and DES use rates (10–40%), and cost inputs, such as stent prices (± 5–10%) and average cost of a hospital bed day (± 5–10%).

## Results

The model estimated that 5674 patients would receive DES in Year 1, increasing to 7890 patients in Year 6, with a total of 40,399 treated patients over a 5-year period starting in 2019 (Table 4). From the perspective of the Australian public healthcare system payer, the base-case model projected a 5-year potential budget saving of $12.1 million in total for a scenario in which all patients eligible for DES implantation were treated with the novel Eluvia DES rather than with the Zilver PTX drug-coated stent. An average of 700 CD-TLRs were saved annually, totalling to a sum of 4198 CD-TLRs potentially avoided from using Eluvia DES compared with Zilver PTX between 2019 and 2024.

**Table 4:** Base-case results from the 5-year Eluvia DES BIM for the Australian national population ($, AUD)

	Year 0 (2019)	Year 1 (2020)	Year 2 (2021)	Year 3 (2022)	Year 4 (2023)	Year 5 (2024)	Total
DES-treated population	**5674**	**6060**	**6474**	**6915**	**7386**	**7890**	**40,399**
Zilver PTX							
Number of CD-TLRs	509	1684	1798	1921	2052	2192	**10,156**
Hospital days for AEs	7082	23,327	24,917	26,617	28,430	30,368	**140,741**
Total cost to the healthcare system (AUD)	$47,538,021	$59,778,429	$63,859,715	$68,211,658	$72,857,805	$77,828,052	**$390,073,681**
Total cost to the hospital system (hospital days) (AUD)	$14,184,422	$46,724,823	$49,907,846	$53,314,184	$56,945,772	$60,826,805	**$281,903,851**
Eluvia DES							
Number of CD-TLRs	257	995	1063	1135	1213	1295	**5959**
Hospital days for AEs	3063	15,958	17,045	18,209	19,449	20,774	**94,498**
Total cost to the healthcare system (AUD)	$46,598,484	$57,831,379	$61,779,920	$65,989,990	$70,484,807	$75,293,271	**$377,977,851**
Total cost to the hospital system (hospital days) (AUD)	$6,134,905	$31,964,449	$34,140,860	$36,471,868	$38,956,238	$41,610,650	**$189,278,972**
Difference							
Number of CD-TLRs avoided	252	689	736	786	839	897	**4198**
Hospital days averted	4019	7369	7872	8409	8981	9594	**46,243**
Total savings to the public healthcare system (AUD)	$939,538	$1,947,050	$2,079,795	$2,221,668	$2,372,998	$2,534,782	**$12,095,830**
Total savings to the hospital (hospital days) (AUD)	$8,049,517	$14,760,374	$15,766,985	$16,842,315	$17,989,534	$19,216,155	**$92,624,879**

Values in bold represent Totals

*AEs* adverse events, *AUD* Australian dollars, *DES* drug-eluting stent, *CD-TLR* clinically driven target lesion revascularisation

From a hospital perspective, the base-case model predicted a 5-year potential budget saving of $92.6 million in total attributable to a reduction in length of hospital stay from subsequent readmissions. A total of 46,243 hospital bed days were made available with Eluvia DES use over the 5-year period, as compared to Zilver PTX use, translating to an average reduction of 7707 days per year in the length of stay.

### Australian State Perspective

The BIM investigated the 5-year budget impact of Eluvia versus Zilver PTX at the Australian state/territory level for DES usage rates from low (10%) to the average base-case rate (28%) (Table 5). At the Australian state/territory level, the potential healthcare system budget savings were driven by the reduction of diagnostic related group (DRG) payments for AEs, as a result of events avoided through use of Eluvia DES. The greatest budget savings (in $ Million, M) were observed in states/territories with highest volumes of DES across a 5-year period: New South Wales and Australian Capital Territory (ACT), $1.430–4.005 M; Victoria, $1.093–3.060 M; Queensland, $0.852–2.386 M; and Western Australia, $0.445–1.247 M. Moreover, these states/territories also showed the greatest potential hospital-level savings due to hospital days avoided for AE: New South Wales and ACT, $10.954–30.670 M; Victoria, $8.370–23.431 M; Queensland, $6.525–18.271 M; and Western Australia, $3.410–9.547 M.

**Table 5:** The net 5-Year BIM results ($, AUD) at the Australian State/Territory level from Eluvia DES use compared with Zilver PTX use, assuming DES use rates between 10.and 28%

	NSW & ACT	VIC	SA	WA	NT	QLD	TAS
Number of patients treated	4788–13,377	3651–10,220	994–2787	1487–4164	142–399	2846–7969	301–845
Number of CD-TLRs avoided	496–1390	379–1062	103–290	155–433	15–41	296–828	31–88
Hospital days for AE avoided	5469–15,312	4179–11,698	1138–3190	1702–4766	162–457	3258–9122	344–967
Total savings to healthcare system (Millions)	$1.430–4.005	$1.093–3.060	$0.297–0.834	$0.445–1,247	$0.042–0.119	$0.852–2.386	$0.090–0.253
Total savings to hospital (Millions)	$10.954–30.670	$8.370–23.431	$2.279–6.390	$3.410–9.547	$0.325–0.915	$6.525–18.271	$0.690–1.937

*ACT* Australian capital territory, *AEs* adverse events, *AUD* Australian dollars, *DES* drug-eluting stent, *CD-TLR* clinically driven target lesion revascularisation, *NSW* New South Wales, *NT* Northern Territory, *QLD* Queensland, *SA* South Australia, *TAS* Tasmania, *VIC* Victoria, *WA* Western Australia

### Sensitivity Analysis

**Fig. 1:**
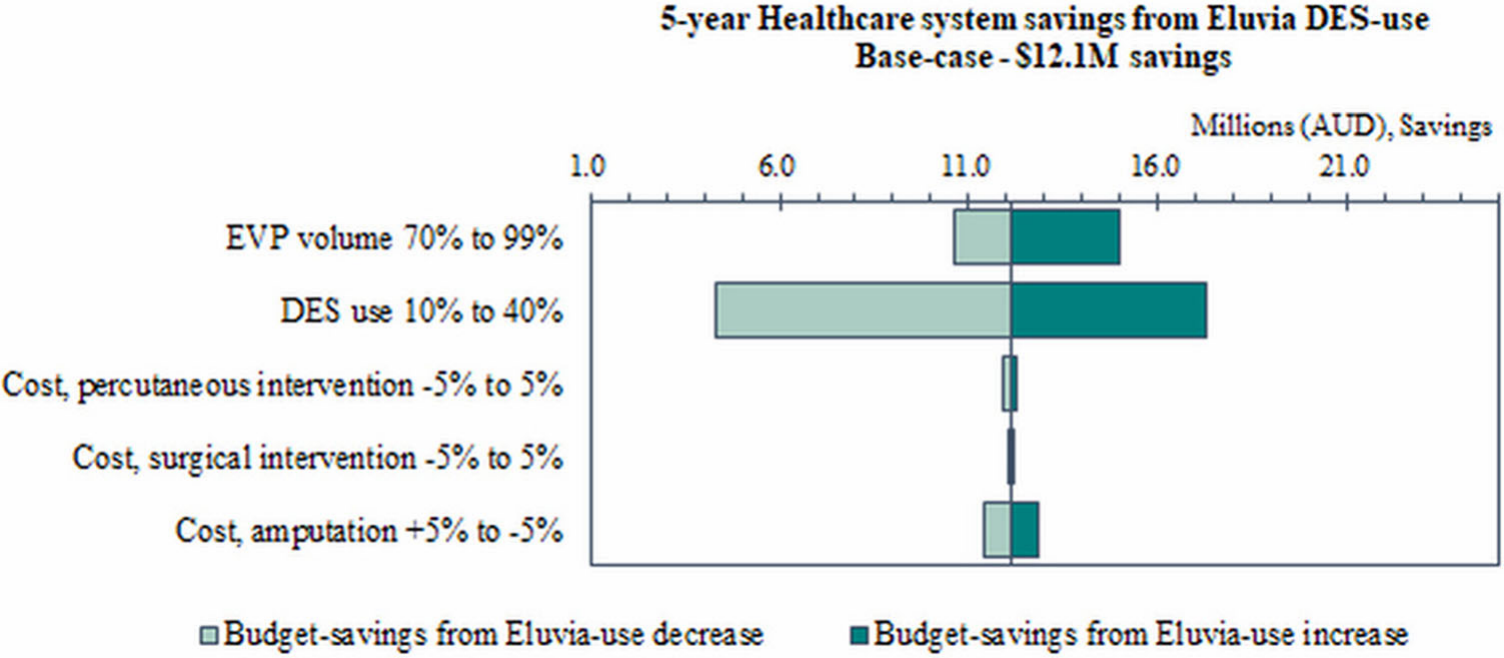
Scenario analyses of net 5-year budget impact ($, AUD) With Eluvia DES versus Zilver PTX only, compared to base-case. *DES* drug-eluting stent, *EVP* endovascular procedure

Scenario analyses were conducted to test the 5-year budget impact of Eluvia DES compared with Zilver PTX, against base-case assumptions (Fig. 1 and Supplementary Table A4). The results illustrate some notable patterns on the budget impact, to variations of frequencies of EVP procedures, DES use, costs, and the price of stents assumed in the base-case. Importantly, the higher the volume of EVP, the greater the potential savings that could be realised from Eluvia DES use when compared with Zilver PTX use. Similarly, a reduction in the overall rate of DES use lowers the magnitude of savings that could be realised from Eluvia use as compared with Zilver PTX. To illustrate, if a DES use rate of 10% was assumed as compared with 28% in the base-case model, the potential healthcare system budget savings reduce to $4.3 million from $12.1million potential savings in the base-case and hospital-level budget savings reduces to $33.1 million from $92.6 million potential savings in the base-case. As such, the potential budget savings that could be realised from use of Eluvia DES were robust to usage rates, suggesting savings could be realised even at low-volume settings.

## Discussion

The budget impact analysis in the present study revealed financial ramifications of using Eluvia DES as compared with Zilver PTX drug-coated stent, for the treatment of patients with symptomatic PAD lesions in the SFA. The 5-year budget impact model which assumed EVP eligibility rate of 80%, and DES use rate ranging from 10 to 28% (superficial femoral artery lesions only) indicated considerable economic impact for the Eluvia DES in the Australian context, underpinned by the superior clinical outcomes observed for this stent in a head-to-head, global RCT [4, 5]. In the IMPERIAL trial, Eluvia DES observed a significant 37% reduction in CD-TLRs at 24 months compared with Zilver PTX; and a 19% reduction in hospital length of stay for adverse events at 12 months, which is the timeframe in which representation for complications, including restenosis is most likely. The model estimated potential budget savings of the magnitude of $4.3–$12.1 million from a healthcare system perspective; and potential savings of $33.1–$92.6 million from a hospital perspective, driven by estimated 1499–4198 fewer CD-TLRs; and 16,515–46,243 fewer hospital days for adverse events, with the use of Eluvia DES than with Zilver PTX across a 5-year period. There was a marked increase in adverse event rates between the first and second cycles, attributed to the inclusion of 24-month outcomes to the first cohort.

From the primary perspective of the public healthcare system payer, the savings from adopting Eluvia DES are considerable, which are driven by reductions in hospitalisations and corresponding activity-based funding cost weights. Delving deeper into how these improved clinical outcomes may impact hospitals, the budget impact or cost savings from hospital perspective is approximately eightfold higher. As the activity-based funding system in Australia reimburses procedures based on their nature rather than on their outcomes, hospitals are incentivised to improve efficiency over time via means such as reduction in the length of stay [14]. The results of this BIM indicated that Eluvia DES offered a clinically effective and cost saving endovascular solution not only to the healthcare system but also to individual hospitals, as savings were realised from both perspectives.

Zilver PTX has been previously compared to BMS and PTA alone, and treatment with Zilver PTX has been shown to be associated with superior 12-month outcomes [1], which were sustained over 5 years [15]. Consequently, Zilver PTX was progressively regarded as the most suitable stent for treating symptomatic PAD. Eluvia DES represents a further advancement in peripheral stenting technology, as highlighted by the results of the IMPERIAL trial. BMSs are still used in practice in Australia, and they remain to be considered as the standard of care for peripheral interventions in some countries. Based on the results of this model and from the previously published budget impact analysis, it could be inferred that transitioning from BMS to Eluvia DES would be associated with considerable cost savings. A budget impact assessment comparing BMS to Eluvia DES should be conducted to address this gap in the literature. Nevertheless, as Zilver PTX was the most likely stent to be replaced by Eluvia DES in Australia, this was deemed the most appropriate comparator.

Both Eluvia and Zilver PTX are approved for symptomatic PAD treatment in Australia and are included on the Prostheses List, which dictates the benefit private health insurances are required to pay for a listed product as part of a hospital treatment. While stents are reimbursed for the same amount at private hospitals, the cost to both public and private hospitals for each stent may fluctuate within 5–10% of the reimbursed amount; therefore, this value was tested in sensitivity analysis. A key assumption in the model is that the price of stents does not change over the time horizon of the model. From the hospital perspective, stent prices did not affect potential savings because savings were driven by observed length of stay for adverse events in the in the IMPERIAL trial (higher for Zilver PTX at 12 months). Post-discharge costs such as outpatient procedure costs and pharmaceuticals costs were not included, as these are reimbursed under funding mechanisms outside the defined scope of this analysis.

The costs related to symptomatic PAD treatment have been previously investigated. De Cock et al. [16] sought to evaluate the financial impact of the implementation of Zilver PTX use as a substitute for BMS in France. The model estimated that staggered introduction of Zilver PTX into the market over 5 years would result in a cumulative saving of €6.8 million to the third-party payer, driven primarily by lower CD-TLR rates. Similarly, the results from this analysis highlight that improved clinical outcomes are associated with considerable cost savings in the treatment of PAD.

Kohn et al. [17] have recently examined the economic burden of hospitalisations for PAD in the USA and reported a median hospital length of stay of 5 days (interquartile range, 3–9) with a total hospital cost of US$15,775, equating to a total annual cost burden of US$6.3 billion. The study determined that hospitalisations due to PAD resulted in a considerable economic burden on the healthcare system, which could be alleviated via improvements in screening and use of effective primary treatment. Accordingly, our study identified the cost savings realised when selecting a treatment option that provides superior clinical outcomes.

The model has several limitations. It was assumed that a patient could not undergo more than one CD-TLR or other revascularisation procedures similar to those in previous models and that any secondary stent placement utilised the same branded stent as in the primary procedure [16]. Model inputs are derived from the IMPERIAL trial; an international multicentre RCT; however, the study did not enrol Australian patients. The IMPERIAL is the only trial that directly compares the safety and efficacy of the Eluvia stent with the established Zilver PTX, which is the alternative stent of choice for treatment in Australia. The IMPERIAL trial was selected as the only source of model input data due to the sound design and methodology, and the high internal validity compared with existing literature. The hospital readmissions and length of stay data were also collected and published, as part of the IMPERIAL trial, making this economic evaluation in effect an extension of the trial. Another trial that enrolled patients from Australia, the MAJESTIC trial [18], a single-arm, multicentre trial of Eluvia in 57 patients with lower-limb ischaemia reported patient baseline characteristics and outcomes at 12 months, with a TLR event rate of 3.5% (2/57), consistent with the IMPERIAL trial [4]. This is comparable to the rate of CD-TLR in the Eluvia arm of the IMPERIAL trial at 12 months (4.5%; 13/287). Furthermore, mortality was not included in the model, as no death was reported in either group in the IMPERIAL trial at 12 months.

Nonetheless, this evaluation has strengths in that it was informed by clinical trial-based outcomes and conservatively estimated the budget savings via inclusion of clinically significant and non-significant readmissions for adverse events as part of the total cost and resource-use equation. For instance, although no significant differences were observed for the rates of major amputation of the target limb in the IMPERIAL trial, which likely occurred as a result of random chance rather than due to device-related reasons, the costs related to the event were considered in this BIM. Additionally, the model conservatively assumed that all readmissions occurred in 24-month time intervals from primary stent intervention and performed no projections beyond this period. It is expected that long-term data from the IMPERIAL trial (up to 5-year follow-up) will be available to inform the model in the future.

## Conclusion

This budget impact model showed considerable potential savings for hospitals and state budgets of the Australian healthcare system, when comparing Zilver PTX to Eluvia as the DES of choice for endovascular interventions. These realised cost savings are underpinned by superior clinical outcomes observed with Eluvia, compared with Zilver PTX, for the treatment of symptomatic lower-limb PAD. The results of this study may yield similar cost savings in other countries; however, country-specific analyses are required.

## Supplementary Information

Below is the link to the electronic supplementary material.

Supplementary file1 (DOCX 54 KB)
