# Supplementary material for: A Budget Impact Model for the use of Drug-Eluting Stents in Patients with Symptomatic Lower-Limb Peripheral Arterial Disease: An Australian Perspective
Source: Cardiovasc Intervent Radiol. 2021 Jun 21;44(9):1375–83. doi: 10.1007/s00270-021-02848-8 (PMC8382623; doi:10.1007/s00270-021-02848-8)
Supplement: Supplementary file 1 — Supplementary file1 (DOCX 54 KB) [file 270_2021_2848_MOESM1_ESM.docx]

Supplementary Data

A Budget Impact Model for the Use of Drug-Eluting Stents in Patients with Symptomatic Lower-Limb Peripheral Arterial Disease: An Australian Perspective

Nishath Altaf, PhD, FRCS, FRACS^1^, Thathya Ariyaratne, PhD^2^, Adrian Peacock, MHTI^3^, Irene Deltetto, MPH^3^, Jad El-Hoss, PhD^2^, Shannon Thomas, FRACS^4^, Colman Taylor, PhD^3,5,6^, Patrice Mwipatayi, MMed (Surg), FCS (SA), FRACS^1^

^1^Department of Vascular Surgery, Royal Perth Hospital, Perth, WA, Australia

^2^Boston Scientific Corporation, Sydney, NSW, Australia

^3^Health Technology Analysts, Sydney, NSW, Australia

^4^Department of Vascular Surgery, Prince of Wales Hospital, Sydney, NSW, Australia

^5^The George Institute for Global Health, Sydney, NSW, Australia

^6^The University of NSW, Sydney, NSW, Australia

Materials and methods

*Epidemiology: Funnel approach [Table 1]*

The funnel approach describes a set of assumptions applied to generate a hypothetical cohort, representative of the total treatment population, through filtration of a broad population.

- Ng et al.[1] observed symptomatic PAD in 6.4% of the population over 50 years of age, which was similar to the reported prevalence rate of symptomatic PAD in Australia and worldwide[2].
- Several reports and trials have indicated that approximately 50% of all patients with symptomatic PAD have lesions in the lower extremities, with 92% of these affecting the SFA[3-6]. As treatment with Eluvia DES for lesions in the proximal popliteal artery is not indicated in Australia, these patients were excluded from the model cohort. The aforementioned statistics were applied to the Australian population aged >50 years, which accounted for approximately 33% of the total population[7].
- It was estimated that 10% of all patients with symptomatic PAD lesions in the SFA would not respond to prescribed lifestyle modifications, exercise regimens, or pharmacologic interventions and would, therefore, be eligible for endovascular intervention[8].
- The actual number of patients who would undergo percutaneous endovascular procedures (EVP; with or without DES) in public hospitals was expected to be lower. As such, these rates were informed by hospital audits conducted by clinical experts at two public hospitals in Western Australia and New South Wales. Audit data from public hospitals indicated that 80–99% of patients with symptomatic PAD underwent an EVP after failed medical therapy; the lower limit of such rate (i.e., 80%) was considered for the base-case cohort of the model.
- While a Canadian health technology assessment informed a DES uptake rate of 28% for peripheral DES[9], it was expected that this would greatly vary according to patient suitability, clinical history, lesion characteristics, and surgeon preference in the Australian setting. Consequently, a DES use rate ranging from 10% to 28% was considered, with the upper limit (i.e., 28%) being included in base-case model. To anticipate an increase in EVP volume due to an ageing population and increasing prevalence of risk factors such as obesity and diabetes, an EVP volume growth rate of 8.42% (estimated through the observed increase in procedures over recent years in Australia) was applied to each year[2]. The total number of patients receiving a DES was compared to the procedural data published by the Australian Institute of Health and Welfare’s National Hospital Morbidity Database to validate the model outcomes[10].

*Costs*

***Healthcare system costs****.* All direct costs related to primary procedure and event rates as observed in the IMPERIAL trial over a 24-month period were included in the model, irrespective of statistical significance. To assess expenditure from a healthcare system perspective, unit costs related to initial and subsequent hospitalisations were identified from the diagnostic codes of the Australian Classification of Health Interventions, Tenth Edition, and the International Classification of Diseases, Tenth Revision, Australian Modification. These codes were mapped to Australian Refined Diagnosis Related Groups (AR-DRG; version 8.0) codes, as determined by the Independent Hospital Pricing Authority. The related national DRG unit prices or average cost weights were identified (Supplementary Table A1)[11]. Each EVP with DES was assigned with a weighted cost average based on the relevant DRG codes and the number of separations for each code, taking into consideration the DRG-reimbursed amount for prostheses, as to not double-count the stent price. Both Eluvia and Zilver PTX are listed on the 2019 Australian Prostheses List at the same benefit/ price level [12]. Although the Prostheses List is only relevant to private payers, this is the only published source of prices for both stents in Australia and was, therefore, used as a proxy for the cost of the device.

Each percutaneous TLR was assigned with the same weighted cost assigned to the primary procedure, irrespective of the type of TLRs, as all percutaneous procedures map to the same DRG codes. The weighted cost for major limb amputations was calculated using DRG codes of both minor and major complexities. It was assumed that DRG codes of minor complexity were associated with percutaneous procedures, whereas DRG codes of intermediate or major complexity were associated with open procedures.

*Incorporating stent price sensitivity*

The average unit cost associated with each DRG encompasses multiple subcategories representing types of costs incurred by the hospitals, such as salaries, imaging, pharmaceuticals, and prostheses. To allow for stent price sensitivity and to avoid double counting when adding the device cost, the line item for prostheses was subtracted from the total DRG cost when calculating the cost for primary stent procedure and TLR with stent insertion. The price of the stent of choice was then added to the updated DRG cost. The same approach was taken for surgical TLR interventions. Unlike the primary intervention, the line item for prostheses was subtracted only for TLR procedures that required a stent placement, but not from the other interventions, as it could not be guaranteed what prosthetic device would be used, if any.

***Hospital costs****.* To capture the potential cost savings not highlighted in the Australian activity-based funding model, the impact of hospital readmissions and hospital days was assessed using the national weighted average cost per hospitalisation and per hospital day (Supplementary Table A2) for all relevant patients in the model[11]. These hospital-level savings are not directly captured within DRG costs and are, therefore, not directly relevant to the national payer. Nevertheless, cost savings related to reduced length of stay and readmissions are realised by the hospital and are valuable in improving the quality of care.

Table A1 DRG codes and weighted cost inputs used in the model

| DRG Code | Description | Average cost | Weighted cost |
| --- | --- | --- | --- |
| **Percutaneous intervention** | |  |  |
| F64C | Skin Ulcers in Circulatory Disorders, Minor Complexity | **$3,009** | **Including prosthesis:**  **$5,894**  **Excluding prosthesis:**  **$5,495** |
| F65B | Peripheral Vascular Disorders, Minor Complexity | **$3,419** |  |
| F14C | Vascular Procedures, Except Major Reconstruction, W/O CPB Pump, Minor Complexity | **$7,508** |  |
| J12B | Lower Limb Procedures W Ulcer or Cellulitis, Minor Complexity | **$10,958** |  |
| J13B | Lower Limb Procedures W/O Ulcer or Cellulitis, Minor Complexity | **$5,656** |  |
| **Surgery/open intervention** | |  |  |
| F64A | Skin Ulcers in Circulatory Disorders, Major Complexity | **$16,347** | **$16,411** |
| F64B | Skin Ulcers in Circulatory Disorders, Intermediate Complexity | **$7,497** |  |
| F65A | Peripheral Vascular Disorders, Major Complexity | **$9,689** |  |
| F14A | Vascular Procedures, Except Major Reconstruction, W/O CPB Pump, Major Complexity | **$33,452** |  |
| F14B | Vascular Procedures, Except Major Reconstruction, W/O CPB Pump, Intermediate Complexity | **$13,877** |  |
| J12A | Lower Limb Procedures W Ulcer or Cellulitis, Major Complexity | **$27,758** |  |
| J13A | Lower Limb Procedures W/O Ulcer or Cellulitis, Major Complexity | **$14,645** |  |
| **Amputation** | |  |  |
| F13A | Amputation, Upper Limb and Toe, for Circulatory Disorders, Major Complexity | **$32,119** | **$35,324** |
| F13B | Amputation, Upper Limb and Toe, for Circulatory Disorders, Minor Complexity | **$14,530** |  |
| F11A | Amputation, Except Upper Limb and Toe, for Circulatory Disorders, Major Complexity | **$68,786** |  |
| F11B | Amputation, Except Upper Limb and Toe, for Circulatory Disorders, Minor Complexity | **$32,712** |  |

Abbreviations: CPB, cardiopulmonary bypass; DRG, diagnosis related code; W, with; W/O, without

Source: Independent Hospital Pricing Authority Cost Data Collection Report Round 21 [13]

Table A2 Notional hospital cost inputs used in the model

| Year | Description | Average cost |
| --- | --- | --- |
| 2014-2015 | Cost per day of hospital stay (NWAU) | $2,003 |

Abbreviations: NWAU, national weighted activity unit

Source: Independent Hospital Pricing Authority [13], Australian Institute of Health and Welfare [14]

Results

Table A3 Base-case results (detailed) from the 5-year Eluvia BIM for the Australian national population

|  | | **Year 0** | **Year 1** | **Year 2** | **Year 3** | **Year 4** | **Year 5** | **Total** |
| --- | --- | --- | --- | --- | --- | --- | --- | --- |
| Total estimated treated population with DES | | 5,674 | 6,060 | 6,474 | 6,915 | 7,386 | 7,890 | 40,399 |
| Zilver PTX | | | | | | | | |
| **Potential no. of adverse events and resource utilisation** | Number of TLRs | 509 | 1,684 | 1,798 | 1,921 | 2,052 | 2,192 | **10,156** |
|  | Number of amputations | 0 | 40 | 42 | 45 | 48 | 52 | **227** |
|  | Number of hospital days for adverse events | 7,082 | 23,327 | 24,917 | 26,617 | 28,430 | 30,368 | **140,741** |
| **Total estimated costs** | Total cost to healthcare system (AUD Million) | $47.54 | $59.78 | $63.86 | $68.21 | $72.86 | $77.83 | **$390.07** |
|  | Total cost hospital system  (AUD Million) | $14.18 | $46.73 | $49.91 | $53.31 | $56.95 | $60.83 | **$281.90** |
| Eluvia DES | | | | | | | | |
| **Potential no. of adverse events and resource utilisation** | Number of TLRs | 257 | 995 | 1,063 | 1,135 | 1,213 | 1,295 | **5,959** |
|  | Number of amputations | 20 | 106 | 113 | 121 | 129 | 138 | **627** |
|  | Number of hospital days for adverse events | 3,063 | 15,958 | 17,045 | 18,209 | 19,449 | 20,774 | **94,498** |
| **Total estimated costs** | Total cost to healthcare system (AUD Million) | $46.60 | $57.83 | $61.78 | $65.99 | $70.48 | $75.29 | **$377.98** |
|  | Total cost hospital system  (AUD Million) | $6.14 | $31.96 | $34.14 | $36.47 | $38.96 | $41.61 | **$189.28** |
| Net annual budget impact from use of Eluvia DES | | | | | | | | |
| **Potential no. of events avoided** | Number of TLRs  avoided | 252 | 689 | 736 | 786 | 839 | 897 | **4,198** |
|  | Number of amputations avoided | (20) | (66) | (71) | (76) | (81) | (86) | **(400)** |
|  | Number of hospital days avoided | 4,019 | 7,369 | 7,872 | 8,409 | 8,981 | 9,594 | **46,243** |
| **Total estimated cost savings** | Total cost savings healthcare system perspective  (AUD Million) | $939.54 | $1.95 | $2.08 | $2.22 | $2.37 | $2.54 | **$12.10** |
|  | Total cost savings hospital perspective  (AUD Million) | $8.05 | $14.76 | $15.77 | $16.84 | $17.99 | $19.22 | **$92.63** |

Abbreviation: TLR, total lesion revascularisation

Table A4 Sensitivity analysis results (detailed*) from the 5-year Eluvia BIM for the Australian national population

| **Scenario** | **Lower limit (Budget-savings from Eluvia-use decrease)** | **Upper limit  (Budget-savings from Eluvia-use increase)** |
| --- | --- | --- |
| **Cost-savings from healthcare system perspective:** | **Base-case savings, $12,095,830** | |
| EVP volume 70% to 99% (base-case: 80%) | $ 10,583,768 | $ 14,968,648 |
| DES use 10% to 40% (base-case: 28%) | $ 4,319,844 | $ 17,279,544 |
| Zilver PTX price -5% to +5% | $ 7,223,841 | $ 16,967,819 |
| Zilver PTX price -10% to +10% | $ 2,351,851 | $ 21,839,809 |
| Eluvia price +5% to -5% | $ 7,261,678 | $ 16,929,982 |
| Eluvia price +10% to -10% | $ 2,427,525 | $ 21,764,135 |
| Cost, percutaneous intervention -5% to 5% | $ 11,922,538 | $ 12,269,122 |
| Cost, percutaneous intervention -10% to10% | $ 11,749,247 | $ 12,442,413 |
| Cost, surgical intervention -5% to 5% | $ 12,017,183 | $ 12,174,477 |
| Cost, surgical intervention -10% to 10% | $ 11,938,535 | $ 12,253,125 |
| Cost, amputation 5% to -5% | $ 11,387,884 | $ 12,803,776 |
| Cost, amputation 10% to -10% | $ 10,679,938 | $ 13,511,772 |
| **Cost-savings from hospital perspective:** | **Base-case savings, $92,624,879** | |
| Cost, average hospital bed day -5% to 5% | $ 87,993,635 | $ 97,256,123 |
| Cost, average hospital bed day -10% to 10% | $ 83,362,391 | $ 101,887,367 |
| DES use 10% to 40% (base-case: 28%) | $ 33,079,622 | $ 132,319,574 |

* *All uncertain variables in the model (that were assumed or derived from public sources) were subjected to scenario analysis. As all clinical inputs were observed from a single source, the IMPERIAL trial, they were considered fixed outcomes, not subjected to sensitivity (or scenario) analysis on this occasion.*

**References**

1. Ng EL, Weiland TJ, Jelinek GA, et al. Prevalence of and risk factors for peripheral arterial disease in older adults in an Australian emergency department*.* *Vascular*. 2013; 22:1-12.

2. Norman PE, Eikelboom JW, and Hankey GJ. Peripheral arterial disease: prognostic significance and prevention of atherothrombotic complications*.* *Med J Aust*. 2004; 181:150-154.

3. Dake MD, Ansel GM, Jaff MR, et al. Paclitaxel-eluting stents show superiority to balloon angioplasty and bare metal stents in femoropopliteal disease: twelve-month Zilver PTX randomized study results*.* *Circ Cardiovasc Interv*. 2011; 4:495-504.

4. Gray WA, Keirse K, Soga Y, et al. A polymer-coated, paclitaxel-eluting stent (Eluvia) versus a polymer-free, paclitaxel-coated stent (Zilver PTX) for endovascular femoropopliteal intervention (IMPERIAL): a randomised, non-inferiority trial*.* *The Lancet*. 2018; 392:1541-1551.

5. Müller-Hülsbeck S, Keirse K, Zeller T, et al. Long-Term Results from the MAJESTIC Trial of the Eluvia Paclitaxel-Eluting Stent for Femoropopliteal Treatment: 3-Year Follow-up*.* *Cardiovasc Intervent Radiol*. 2017; 40:1832-1838.

6. Zeller T. Current state of endovascular treatment of femoro-popliteal artery disease*.* *Vasc Med*. 2007; 12:223-234.

7. Australian Bureau of Statistics. *3235.0 - Regional Population by Age and Sex, Australia, 2017*. Canberra, Australia: Australian Government; 2018. Published September 28, 2018.

8. Gerhard-Herman MD, Gornik HL, Barrett C, et al. 2016 AHA/ACC Guideline on the Management of Patients With Lower Extremity Peripheral Artery Disease: Executive Summary: A Report of the American College of Cardiology/American Heart Association Task Force on Clinical Practice Guidelines*.* *Circulation*. 2017; 135:e686-e725.

9. Medical Advisory Secretariat. *Stenting for peripheral artery disease of the lower extremities: an evidence-based analysis.* 2010. Ontario Health Technology Assessment Series.

10. Australian Institute of Health and Welfare. *Procedures Data Cubes*. Canberra, Australia: Australian Government; 2019. <https://www.aihw.gov.au/reports/hospitals/procedures-data-cubes/contents/data-cubes>. Updated May 23, 2019. Accessed June 19, 2019

11. Independent Hospital Pricing Authority. *National Hospital Cost Data Collection, Public Hospitals Cost Report, Round 21 (Financial Year 2016–17)*. Darlinghurst, Australia: 2019. <https://www.ihpa.gov.au/publications/national-hospital-cost-data-collection-report-public-sector-round-21-financial-year>. Published March 12, 2019. Accessed June 19, 2019

12. Department of Health. *Prostheses List - Part A*. Canberra, Australia: Australian Government; 2019. <https://www1.health.gov.au/internet/main/publishing.nsf/Content/health-privatehealth-prostheseslist.htm>. Accessed June 19, 2019

13. Independent Hospital Pricing Authority, National Hospital Cost Data Collection Cost Report: Round 21 Financial Year 2016-17. 2019, IHPA: Sydney.

14. Australian Institute of Health and Welfare, Hospital performance: Costs of acute admitted patients in public hospitals from 2012–13 to 2014–15., i*n Cat. no. HPF 18.* 2018, AIHW: Canberra.
